# CRISPR Base Editing to Create Potential Charcot–Marie–Tooth Disease Models with High Editing Efficiency: Human Induced Pluripotent Stem Cell Harboring *SH3TC2* Variants

**DOI:** 10.3390/biomedicines12071550

**Published:** 2024-07-12

**Authors:** Camille Loret, Amandine Pauset, Pierre-Antoine Faye, Valérie Prouzet-Mauleon, Ioanna Pyromali, Angélique Nizou, Federica Miressi, Franck Sturtz, Frédéric Favreau, Béatrice Turcq, Anne-Sophie Lia

**Affiliations:** 1 University of Limoges, NeurIT UR 20218, GEIST Institute, F-87000 Limoges, France; pierre-antoine.faye@unilim.fr (P.-A.F.); ioanna.pyromali@unilim.fr (I.P.); angelique.nizou@unilim.fr (A.N.); federica.miressi@unilim.fr (F.M.); franck.sturtz@unilim.fr (F.S.); frederic.favreau@unilim.fr (F.F.); anne-sophie.lia@unilim.fr (A.-S.L.); 2 University of Bordeaux, CRISP'edit, TBMCore UAR CNRS 3427, US Inserm 005, F-33000 Bordeaux, Francevalerie.prouzet-mauleon@u-bordeaux.fr (V.P.-M.); beatrice.turcq@u-bordeaux.fr (B.T.); 3 University of Bordeaux, Modeling Transformation and Resistance in Leukemia, BRIC Inserm U1312, F-33000 Bordeaux, France; 4 CHU Limoges, Department of Biochemistry and Molecular Genetics, F-87000 Limoges, France; 5 CHU Limoges, Department of Bioinformatics, F-87000 Limoges, France

**Keywords:** hiPSCs, CRISPR-Cas9, disease cellular model, Charcot–Marie–Tooth (CMT), *SH3TC2*, HEK293-T

## Abstract

Human induced pluripotent stem cells (hiPSCs) represent a powerful tool to investigate neuropathological disorders in which the cells of interest are inaccessible, such as in the Charcot–Marie–Tooth disease (CMT), the most common inherited peripheral neuropathy. Developing appropriate cellular models becomes crucial in order to both study the disease’s pathophysiology and test new therapeutic approaches. The generation of hiPS cellular models for disorders caused by a single nucleotide variation has been significantly improved following the development of CRISPR-based editing tools. In this study, we efficiently and quickly generated, by CRISPR editing, the two first hiPSCs cellular models carrying alterations involved in CMT4C, also called AR-CMTde-*SH3TC2*. This subtype of CMT is associated with alterations in the *SH3TC2* gene and represents the most prevalent form of autosomal recessive demyelinating CMT. We aimed to develop models for two different *SH3TC2* nonsense variants, c.211C>T, p.Gln71* and the most common AR-CMTde-*SH3TC2* alteration, c.2860C>T, p.Arg954*. First, in order to determine the best CRISPR strategy to adopt on hiPSCs, we first tested a variety of sgRNAs combined with a selection of recent base editors using the conveniently cultivable and transfectable HEK-293T cell line. The chosen CRISPR base-editing strategy was then applied to hiPSCs derived from healthy individuals to generate isogenic CMT disease models with up to 93% editing efficiency. For point mutation generation, we first recommend to test your strategies on alternative cell line such as HEK-293T before hiPSCs to evaluate a variety of sgRNA-BE combinations, thus boosting the chance of achieving edited cellular clones with the hard-to-culture and to transfect hiPSCs.

## 1. Introduction

There are currently more than 6053 rare diseases annotated with prevalence or incidence information in the Orphanet database, and only 5% are estimated to have at least one approved treatment (The Lancet Diabetes & Endocrinology, 2019). With the continuously growing number of diagnosed genetic disorders, it is crucial to develop appropriate models to study their pathophysiology and to test new therapeutic approaches. Neurological disorders pose additional challenges due to the difficulty of accessing patient cells, further underscoring the need for suitable cellular models.

Human induced pluripotent stem cells (hiPSCs) have emerged as a powerful tool to model diseases, including neurological disorders [1]. In the right conditions, hiPSCs can be differentiated, for instance, in neuronal cells such as motoneurons and Schwann cells [2,3,4]. However, the reprogramming process to obtain hiPSCs from patients is time-consuming and requires a surgical procedure (skin biopsy).

In the last few years, the development of genome editing technologies based on programmable nucleases has made considerable advances. With the CRISPR-Cas9 methods [5,6], the precise genomic edition of eukaryotic cells is now possible [7,8]. The downside of this now well-known genome editing method lies in the requirement of double-strand DNA breaks (DSB) as an initiation step, which often leads to major alterations in the genome, mostly indels but also megabase-scale chromosomal truncations [9]. As an alternative, several academic groups developed and engineered the “base editing” CRISPR strategy [10]. Without the potentially harmful requirement of DSBs or the inefficient HDR-mediated mechanisms, base editing largely facilitates the creation of cellular models in the case of point mutations. In this system, cytidine base editors (CBEs) and adenine base editors (ABEs), respectively, induce genomic C-to-T and A-to-G substitutions [10]. More recently, various works have permitted the limitations of the original base editing strategy to be solved by including a smaller window activity [11] and a relaxed protospacer adjacent motif (PAM) with close to no sequence specificity [12].

While the use of base editing and engineered Cas9 allows targeting nearly the entire genome with high editing efficiency, finding the optimal combination of Base Editor (BE) and single guide RNA (sgRNA) to allow the best editing conditions is a hazardous path. For instance, the diversity of the genomic environment [13], as well as the epigenetic states [14], could hamper the efficiency of the process. Creating models efficiently is even more complex with hiPSCs, which are time-consuming and costly to culture, transfect, and screen. Therefore, assessing different BE-sgRNA combinations before hiPSCs engineering seems to be a requirement.

The Charcot–Marie–Tooth disease (CMT), with its complex genetic landscape, stands among neurological disorders where research faces significant hurdles due to the need for appropriate models. With a prevalence of 1 in 2500 people worldwide [15], CMT is the most common form of inherited peripheral neuropathy, characterized by symptoms affecting both sensory and motor nerves [16]. CMT is a genetically and clinically highly heterogeneous group of disorders caused by inherited or de novo alterations affecting a panel of specific genes [17] (https://neuropathybrowser.zuchnerlab.net/#/, accessed on 7 November 2022). The autosomal recessive demyelinating form of CMT, CMT4C, also called AR-CMTde-*SH3TC2*, caused by alterations in *SH3TC2*, has been reported to be the most prevalent autosomal recessive form, with frequencies exceeding 20% [18,19,20,21].

Currently, no human in vitro models have been used to study the endogenous expression of the SH3TC2 protein, which includes two Src homology 3 (SH3) domains followed by ten tetratricopeptide repeat (TPR) motifs. For many forms of Charcot–Marie–Tooth disease (CMT), such as AR-CMT caused by mutations in the SH3TC2 gene, research into the underlying mechanisms and potential therapeutic approaches now requires more advanced models. hiPSCs CRISPR-engineered models could fulfil this role.

Herein, we generated the first hiPSCs models for the AR-CMTde-*SH3TC2* with high efficiency. We first utilized CRISPR-Cas9 base editing on the easy-to-grow and transfected HEK-293T cell line to optimize the editing strategy for each alteration. We then applied the optimized strategy on control hiPSCs and obtained a high editing efficiency with up to 93% edited clones at the position of interest. In one model, we targeted the most frequent alteration (c.2860C>T; p.Arg954*) found in AR-CMTde-*SH3TC2* patients [21]. This variation, corresponding to half of the observed variants in *SH3TC2* [22], is located in the TPR motifs area and leads to a TGA non-sense codon. Most of the other variations have a very low allele frequency (around 1%); we chose to generate the variation (c.211C>T; p.Gln71*) since it is located close to the SH3 domains and leads to a different non-sense codon TAG [23].

## 2. Materials and Methods

### 2.1. Editing Strategy: Plasmids and Oligonucleotides

Plasmids pCAG-CBE4max-SpRY-P2A-EGFP (RTW5133) and pCAG-CBE4max-SpG-P2A-EGFP (RTW4552) were a gift from Benjamin Kleinstiver (Addgene plasmid # 139999; http://n2t.net/addgene:139999, accessed on 15 August 2022; RRID:Addgene_139999 and Addgene plasmid # 139998; http://n2t.net/addgene:139998, accessed on 15 August 2022; RRID:Addgene_139998, respectively). Plasmid YE1-BE4max-NG was a gift from David Liu (Addgene plasmid # 138159; http://n2t.net/addgene:138159, accessed on 15 August 2022; RRID:Addgene_138159).

The gRNA cloning cassette (U6-promoter-BbsI-gRNA scaffold) was amplified from pX330-U6-Chimeric_BB-CBh-hSpCas9 plasmid, a gift from Feng Zhang (www.addgene.org/42230/, accessed on 15 August 2022) and inserted in pUC19 plasmid, a gift from Joachim Messing (Addgene plasmid # 50005; http://n2t.net/addgene:50005, accessed on 15 August 2022; RRID:Addgene_50005), between the XbaI-KpnI sites. Oligonucleotides corresponding to spacers of chosen gRNA were synthetized by Eurogentec (Seraing, Belgium) and cloned in this pUC19gRNA expression plasmid at BbsI sites as described in Cong et al. [24].

According to the following criteria, different sgRNA oligonucleotides were designed to encompass either *SH3TC2* c.211 or c.2860: (1) the position of the alteration has to be in the center of the activity window (between the 4th and the 8th nucleotide), (2) the appropriate PAM must be at position 21 to 23 at the 5’end, and (3) the sgRNA must be 20 nucleotides long.

### 2.2. Cell Culture and Transfection of HEK-293T

Human embryonic kidney 293T cell lines (HEK-293T) (ATCC, Manassas, VA, USA) were cultured in Dulbecco’s Modified Eagle’s Medium (DMEM) (Gibco, Thermo Fisher SCIENTIFIC, Waltham, MA, USA), supplemented with 10% (*v*/*v*) fetal bovine serum (FBS) (Gibco, Thermo Fisher SCIENTIFIC), 10% Glutamine (Gibco, Thermo Fisher SCIENTIFIC) and 1% penicillin/streptomycin (Gibco, Thermo Fisher SCIENTIFIC).

HEK-293T cells were transfected with different combinations of BE and sgRNA expressing plasmids using the Phosphate Calcium transfection methods. The next day, GFP (Green Fluorescent Protein) fluorescence was assessed by microscopy (ZOE Fluorescent Cell Imager, Bio-Rad, Hercules, CA, USA) in order to monitor the transfection efficiency.

### 2.3. Genotyping of HEK-293T Cells

Two to three days post-transfection, HEK-293T cells were pelleted after trypsinization (Gibco, Thermo Fisher SCIENTIFIC). The pellet was resuspended with lysis buffer of PHIRE Tissue Direct PCR Master mix (Gibco, Thermo Fisher SCIENTIFIC), kept at RT (room temperature) for 2 min and heated at 95 °C for 2 min.

PCR was performed using 1 µL of this crude lysate as a template on the Bio-Rad Thermal Cycle T100 (Bio-Rad, Hercules, CA, USA) using PHIRE Tissue Direct PCR Master mix (Gibco, Thermo Fisher SCIENTIFIC). PCR products were Sanger sequenced by Eurofins (Ebersberg, Germany). Results from Sanger sequencing were treated with the SnapGene Viewer 4.3.11 software (Dotmatics, Boston, MA, USA, version 5.3.2).

The Allele-Specific PCR (AS-PCR) method, previously described by Jiang et al., was used to estimate the editing efficiency [25]. It was performed using 1 µL of this crude lysate as a template. Two sets of primers were used to confirm both the ratio of wild-type (WT) and edited DNA in the pool of cells: (1) a common primer to both AS-PCR that perfectly hybridizes to a non-mutated area; (2) a second primer that gives the specificity and whose base placed in 3’ is either the base found in the WT or in the mutated allele. To increase the specificity, as one mismatch would not be enough to discriminate between WT and edited allele, an additional mismatch at position −3 starting from the last base was deliberately introduced, as described by Jiang et al. [25]. The AS-PCR steps were performed on the CFX Connect, Real-time Bio-Rad System using qPCR Brilliant III Ultra-Fast SYBR Green (Agilent Technologies, Santa Clara, CA, USA). Experiments were at least performed in duplicate. The AS-PCR results were transcribed in editing efficiency (%) by applying the «2e- ∆Ct» formula. The primers used for PCR and AS-PCR amplification are listed in Table S5.

### 2.4. Culture and Nucleofection of hiPSCs

The wild-type hiPSC clone supplied arises from a healthy 24-year-old male donor. Previous investigations have allowed us to determine that this individual did not carry any peripheral neuropathy or any alterations in *SH3TC2*. The clone (reprogramming carried out in 2020) was obtained as previously described [26].

hiPSCs were maintained on feeder-free culture on a layer of diluted Matrigel (Corning Incorporated, New York, NY, USA) in mTeSR™Plus medium (Stemcell Technologies, Grenoble, France) at 37 °C, 5% CO_2_. The culture medium was changed every other day. Passages were carried out every 5 to 8 days using StemMACS Passaging solution XF (Miltenyi Biotec, Bergisch Gladbach, Germany). Cells in passages 15 to 25 were used in this study. Two passages after thawing, the healthy donor hiPSCs were cultured up to 70–80% confluence in a Matrigel-coated six-well culture plate. Two hours prior to nucleofection, the culture medium was changed and supplemented with Rho Kinase inhibitor Y27632 (10 μM) (Miltenyi Biotec). Briefly, hiPSCs were washed with 1X-PBS and harvested with accutase (Stemcell Technologies) in a humidified incubator at 37 °C for 10 to 12 min before nucleofection. An amount of 1 × 10^6^ cells per condition were pelleted and resuspended with the supplemented nucleofection solution (Human Stem Cell Nucleofector™ Kit, 1 #VPH-5012, Lonza, Basel, Switzerland), 2 µg of the base editor plasmid, and 2 µg of the sgRNA plasmid according to the manufacturer’s recommendations. Nucleofection was performed immediately after, using the B-016 program of the Nucleofector^®^ 2b Device (Lonza). This program was priorly identified as the most suitable one. The transfected cells were then seeded into a 12-well plate coated with Matrigel and cultured in mTeSR™1-CloneR2 (10%) (Stemcell Technologies) for 48 h. For each condition, a control without plasmid was carried out.

### 2.5. Flow Cytometry: hiPS Cells Sorting

The nucleofected hiPSCs were dissociated with Accutase 48 h after seeding. The solution was centrifuged, and the pellet was then resuspended in 1X-DPBS-mTeSR™1-CloneR2. The sgRNA plasmid contains an EGFP sequence, allowing us to easily select the transfected cells by cell sorting (BD Biosciences, Franklin Lakes, NJ, USA; FACS ARIA III; FITC). GFP-positive cells were sorted using the most stringent sorting mode (single cell) and the 70 µm nozzle. Selected cells were then seeded in a 96-well plate (1 cell/well) containing mTeSR™Plus medium supplemented with CloneR2 and 0.1% Gentamicin (Gibco, Thermo Fisher SCIENTIFIC). Three 96-well plates were used for clonal expansion.

### 2.6. hiPSC Clones Screening

Genotyping of hiPSC clones that had grown after cell sorting was carried out when passaging cells from the 48-well plate to the 24-well plate. Cells were dissociated with StemMACS Passaging solution XF (RT). Half of the scrapped cells were used for passaging; the other half was collected and pelleted (5 min, 140 g). The supernatant was withdrawn while the pellet was resuspended with a homemade Lysis buffer [27]. The mixture was kept at 56 °C for 1 h, followed by 95 °C for 15 min.

PCR was performed using 1 µL of the lysis mixture as a template. The same primers used to genotype HEK-293T cells were used for hiPSCs (Table S5). The PCR steps were performed on a T100 Thermal Cycler using the TransTaq DNA Polymerase High Fidelity (HiFi) (TransGen Biotec, Beijing, China).

Amplification products were cleaned up using the EasyPure PCR Purification Kit (TransGen Biotec) and were then submitted to Sanger sequencing using the BigDye Terminator v1.1 Cycle sequencing Kit (Gibco, Thermo Fisher SCIENTIFIC). The extension products were cleaned up with the DyeEx 2.0 Spin Kit (Qiagen, Hilden, Germany) and then separated by capillary electrophoresis on a 3130xl Genetic Analyser sequencer (Gibco, Thermo Fisher SCIENTIFIC). The obtained sequences were analyzed with the SnapGene Viewer 4.3.11 software. To double-check the base editing efficiency, all the steps presented in Section 2.6 have been replicated for positive clones when passaging cells from the 24-well plate to the 12-well plate.

### 2.7. Bystander Editing Analysis

To evaluate the editing efficiency distribution observed along the target sequence, for each clone, homozygote nucleotides were counted as two, and heterozygous one was counted as one. This number was normalized on the total number of alleles analyzed.

### 2.8. Off-Target Site Selection and Amplicon Design

For both models, potential off-target sites were determined using the CRISPOR tools (Concordet and Haeussler, 2018 [28]) (Version 4.99) with a threshold of four mismatches. The MIT off-target score, as well as the CFD Off-target score, were used to discriminate them. Ten different sites displaying lower mismatch count and/or the highest rate in either score (when available) were investigated (CFD Off-target Score at least >0.02) [11] (Tables S3 and S4). For all sequences, the corresponding genomic regions were amplified, then the amplicons were sequenced to investigate any off-target editing for those areas (Tables S6 and S7). All analyses were performed using the hg19 reference genome.

### 2.9. hiPSCs Characterization

According to the official guidelines of the International Society for Stem Cell Research, the following experiments have been performed to characterize the edited hiPSCs clones. We examined the morphology of the colonies and performed immunostaining analysis for pluripotency markers (Table S8). The presence of alkaline phosphatase was confirmed using the SIGMAFAST™ BCIP^®^/NBT substrate (Sigma-Aldrich, Merck, Saint-Louis, MO, USA). Additionally, we conducted an array of Comparative Genomic Hybridization (aCGH) analyses for both the homozygote V1 and V2 clones in comparison to the parental control hiPSC genome to verify the absence of significant genomic copy number variations (CNVs). Furthermore, the absence of mycoplasma contamination was verified using the MycoAlert™ PLUS Mycoplasma Detection kit (Lonza).

## 3. Results

### 3.1. Base Editing: How to Quickly Determine the Best CRISPR Strategy Using HEK-293T Cells

**Figure 1:**
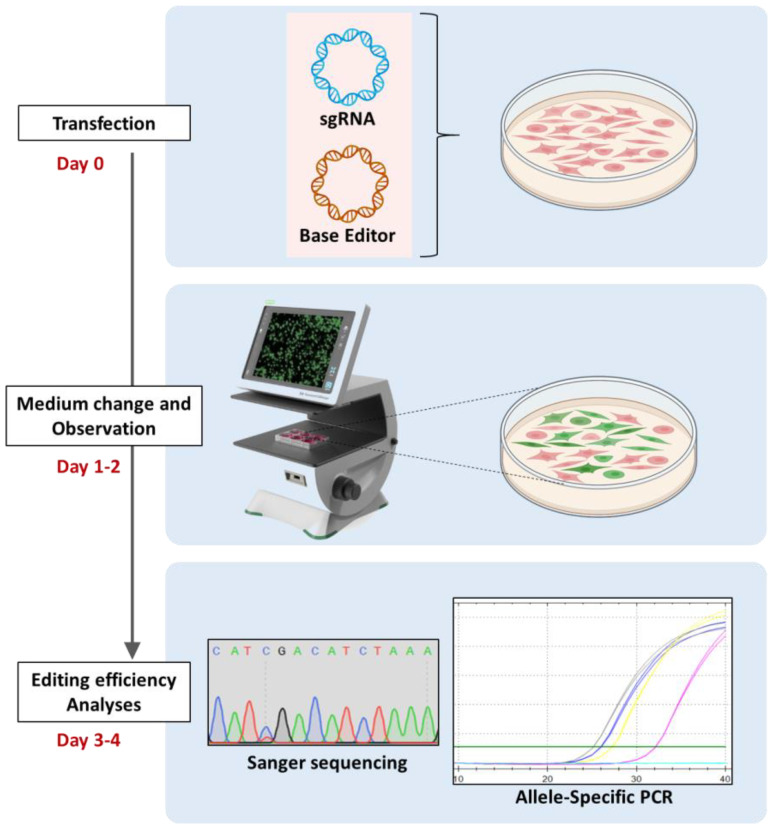
**Base editing strategy assessments in HEK-293T cells.** Schematic illustration depicting the gene-editing strategy workflow on HEK293-T cells. On day 0, HEK293-T cells are transfected with a combination of a Base Editor (BE) and a sgRNA using CaCl_2_ as a transfecting agent. Cells are then seeded at 4 × 10^6^ cells per well in a six-well plate. One to two days later, the amount of transfected cells is assessed by microscopy, and green cells represent GFP^+^ cells. Then, on the 3rd and 4th day, Sanger sequencing and allele-specific PCR are performed. The blue-wired circle represents the sgRNA plasmid, and the brown wired circle represents the base editor plasmid. The transfected agent is symbolized as an orange oval.

Base editing emerges as a highly effective strategy for creating new hiPSC models that only vary by one single base. However, it is important to note that there are often multiple combinations of base editor (BE) and guide RNA (sgRNA) that can be used for this purpose. As hiPSCs are both hard to transfect and to culture, we chose not to evaluate the editing feasibility with this cell type, as it would be time and money-consuming. We decided to use the convenient, economical, cultivable, and transfectable HEK-293T cells as a first step to test different CRISPR-Cas9 base editing strategies. HEK-293T cells appear to be the prime choice, as this cell line is economical, cultivable, and easy to transfect. We opted for two main experiments on the CRISPR-cell lysates: (1) Sanger sequencing to first roughly visually evaluate the editing, and (2) allele-specific PCR (AS-PCR) to assess the ratio of editing. AS-PCR was performed using two sets of primers to estimate both the ratio of wild-type and edited DNA in the cell pool. With this strategy, only four days are necessary to evaluate various combinations of sgRNA-BE and to determine which one was the safest choice in order to continue on hiPSCs (Figure 1).

### 3.2. Assessment of Base Editing Efficiency

**Figure 2:**
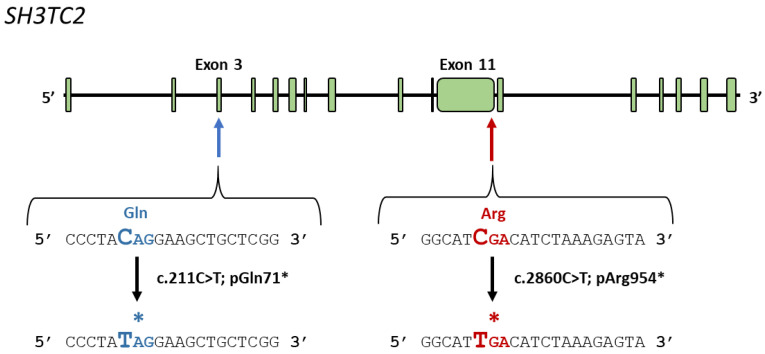
**Schematic illustration of the relevant regions of the *SH3TC2* locus.** The area encompassing the positions of interest are pointed by a blue (c.211C>T; pGln71*) and a red arrow (c.2860C>T; pArg954*). The nucleotides depicted in the figure represent the specific targets for the sgRNAs used in CRISPR-mediated cell editing. The codons undergoing the variations are indicated in bold color, and a mutated couple of nucleotides are represented by bigger letters. The star indicates a stop codon.

To illustrate our approach, we present here the creation of the first hiPSC models harboring a single base variation in SH3TC2. We targeted two specific regions of *SH3TC2*: exon 3, where we aimed to edit c.211C>T; p.Gln71* and exon 11, where we intended to perform the c.2860C>T; p.Arg954* edit (Figure 2).

**Figure 3:**
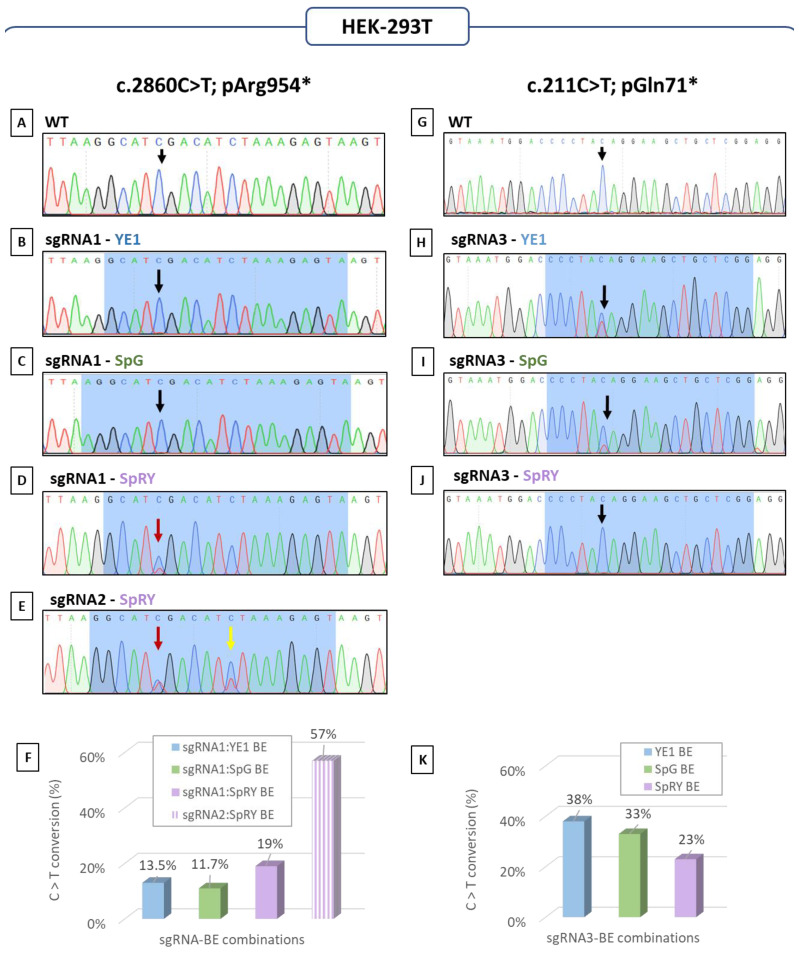
**Genotyping assays to establish the optimal base editing strategy using HEK-293T models.** Sanger sequencing of the region of interest *SH3TC2*-c.2860C in HEK-293T before transfection (**A**) wild-type profile (WT), after transfection of sgRNA1 associated with (**B**) YE1 BE, with (**C**) SpG BE or with SpRY BE (**D**) and after transfection of SpRY BE associated with (**E**) sgRNA2. The black arrow points to the position of interest; the red one is the detectable base editing, and the yellow one is the detectable bystander editing. (**F**) The AS-PCR results in HEK-293T are expressed in editing efficiency (%). sgRNA1 results are represented by plain color, and the sgRNA2 result is represented by vertical stripes. Sanger sequencing of the region of interest *SH3TC2*-c.211 in HEK-293T before transfection (**G**) wild-type profile (WT), and after transfection of sgRNA3 associated with (**H**) YE1 BE, with (**I**) SpG BE or with SpRY BE (**J**). The black arrow points to the position of interest, the red one to the detectable base editing, and the yellow one to the detectable bystander editing. (**K**) The AS-PCR results in HEK-293T are expressed in editing efficiency (%).

For the *SH3TC2*-c.2860C>T-p.Arg954* alteration, a first guide with the targeted nucleotide in position 5 (sgRNA1) was used combined with three of the most recent BE: YE1-BE4max-NG (YE1), pCAG-CBE4max-SpG-P2A-EGFP (SpG) and pCAG-CBE4max-SpRY-P2A-EGFP (SpRY) (Table S1). On the pool of HEK-293T cells, the intended editing event was observed by Sanger sequencing, with the SpRY BE in comparison to the wild-type profile (WT) (Figure 3A,D), but not with the two other base editors (Figure 3A–C). A second sgRNA guide (sgRNA2) with the targeted nucleotide in position 6, presenting a different PAM than the sgRNA1, was suitable but only with the SpRY BE (Table S1). Using this guide, we used Sanger sequencing for the high level of editing (Figure 3A,E). Despite the Sanger sequencing suggesting no editing with certain sgRNA-base editor combinations, AS-PCR analysis showed that all combinations induced the intended editing event, with efficiencies estimated from 11.7% to 19% for sgRNA1-BE combinations and reaching 57% for sgRNA2 combined with the SpRY (Figure 3F). Consistent with the Sanger sequencing results, the combination of sgRNA2 with the SpRY base editor exhibited the highest editing efficiency, with a threefold increase compared to sgRNA1. However, bystander editing was also observed with sgRNA2 (Figure 3E).

For the second alteration, *SH3TC2*-c.211C>T-p.Gln71*, only one guide was suitable for base editing (sgRNA3). SgRNA3 was used in combination with the same three BE previously mentioned (Table S2). According to the Sanger sequencing, the YE1 BE seemed to offer the highest editing efficiency, followed by the SPG BE (Figure 3H,I). However, no editing was discernible in the Sanger sequencing data when employing the SpRY BE (Figure 3J). These results were further validated through AS-PCR analysis, which revealed that the lowest efficiency was obtained with the SpRY BE (23%), while the highest one was achieved with the YE1 BE (38%) (Figure 3K). SpG BE associated with sgRNA3 also allowed a good rate of editing (33%) (Figure 3K).

We showed here that, in only a few days, we were able to evaluate a variety of CRISPR base editing strategies on HEK-293T cells. Moreover, these results indicate that there is not an ideal BE to obtain a Cytosine (C) to Thymine (T) (C>T) edit and that it is quite important to test various combinations of sgRNA + BE to establish the preferred strategy.

### 3.3. Attempt for Generating SH3TC2 Alterations: From HEK-293T Cells to hiPSCs

**Figure 4:**
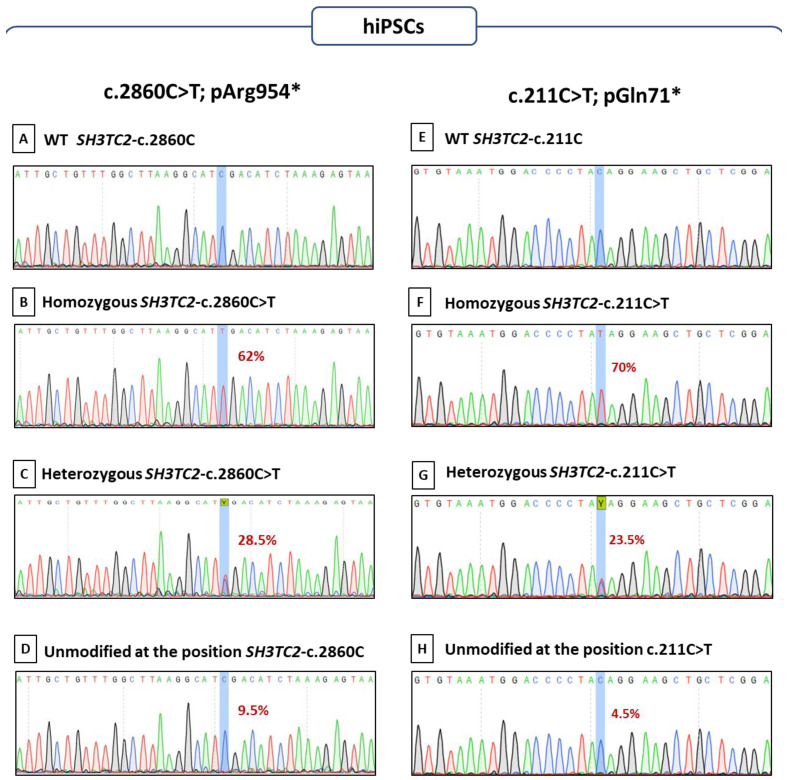
**Sanger sequencing of the region of interest in hiPSCs**: for the *SH3TC2*-c.211C>T-p.Gln71* alteration (**A**) wild-type hiPSCs (WT), (**B**) homozygous clone, (**C**) heterozygous clone, and (**D**) unmodified clone and for the *SH3TC2*-c.2860C>T-p.Arg954* alteration (**E**) wild-type hiPSCs (WT), (**F**) homozygous clone, (**G**) heterozygous clone and (**H**) unmodified clone. The cytosine of interest is highlighted in blue. The ratios of C>T edition at the position of interest are specified in bold red.

Following the successful editing on HEK-293T cells, we applied the sgRNA2 associated with SpRY BE to generate the first alteration, *SH3TC2*-c.2860C>T-p.Arg954*, on control hiPSCs. At the position of interest “c.2860”, of the 21 resulting subclones, 13 homozygotes (62%) and six heterozygotes (28.5%) edited clones were obtained (Figure 4A–C). Only two clones (9.5%) stayed unedited at position c.2860 (Figure 4A compared to Figure 4D).

The same strategy was then applied on the second alteration *SH3TC2*-c.211C>T-p.Gln71*. Despite the higher editing rate allowed by the YE1 base editor (38%), we selected the SpG BE due to the advantage of incorporating a GFP sequence for selection and its comparable editing efficiency (33%). At the position of interest, “c.211”, of the 47 resulting subclones, 33 homozygotes (70%) and 11 heterozygotes (23.5%) edited clones were obtained (Figure 4E compared to Figure 4F,G). Only two clones (4.5%) stayed unmodified as they still harbored the original pattern (Figure 4E compared to Figure 4H), and one clone carried a transversal alteration (*SH3TC2*-c.211C>G, p.Gln71Glu).

To summarize, we notably obtained 90.5% and 93% of on-target activity, allowing the correct editing at the positions of interest in at least one allele for *SH3TC2*-c.2860C>T-p.Arg954* and *SH3TC2*-c.211C>T-p.Gln71* respectively on our hiPSC sorted clones. These remarkable results were obtained in one trial using the combination of plasmid and sgRNA chosen with the preliminary HEK-293T cells experiments. For each alteration, we observed the noteworthy acquisition of a substantial quantity of homozygous clones, which are fundamental for accurately replicating the disease.

### 3.4. Bystander Editing

**Figure 5:**
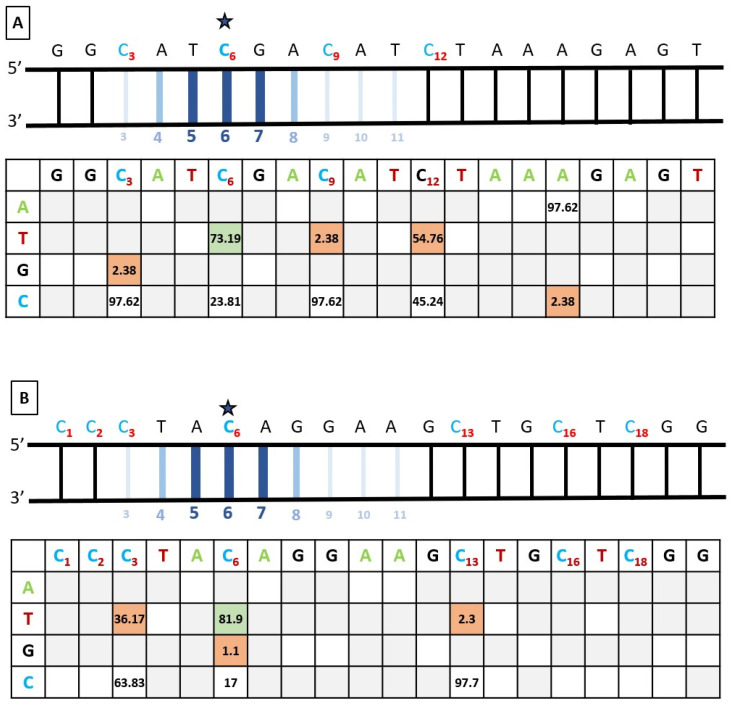
**An extended activity window was** observed after base editing in (**A**) *SH3TC2*-c.2860C>T-p.Arg954* hiPSCs and (**B**) *SH3TC2*-c.211C>T-p.Gln71* hiPSCs. In the upper part, the theorical editing window is represented and displayed by the base editor. All cytosines are colored in blue with positions indicated by bold red subscripted numbers. A blue star pinpoints the cytosine of interest. Hydrogen bonds are represented by large dark blue lines in the center of the activity window and with a narrower and lighter color when surrounding. In the lower part of the panels, the mean editing efficiency distribution observed along the target sequence is represented in n = 21 biologically independent samples (for (**A**)) and in n = 47 biologically independent samples (for (**B**)). The edit of interest is represented with a green background, and the unintended edit is represented by an orange one. White and grey backgrounds, respectively, represent natural nucleotides with no editing. For both experiments, the editing window was located between the 4th and the 8th nucleotide.

Bystander editing, often encountered when performing base editing, could pose significant challenges. For both alterations, the editing window was located between the 4th and the 8th nucleotide, and nucleotides 5 to 7 had the highest chance of editing. Still, three C at the 3rd, 9th, and 12th positions and six C at the 1st, 2nd, 3rd, 13th, 16th, and 18th positions were respectively comprised in the sgRNA2 and sgRNA3 (Figure 5A,B). They could potentially be subject to cytosine base editing, as the activity window does not follow a rigorous pattern.

As expected, when using the sgRNA2 in hiPSCs, we observed a C>T editing in position 9th, very close to our nucleotide of interest (6th position). However, this bystander editing was observed in only 2.38% of our subclones (Figure 5A). Surprisingly, it is at position 12 that an edition from C to T was observed more frequently in 12 of our 21 clones. In addition to the expected C>T editing, a Cytosine (C) to Guanine (G) (C>G) alteration was observed in one subclone at the 3rd position and an A>C modification in one subclone at position 16 (Figure 5A). Still, the on-target activity was greater, with more than 93% of the C>T ratio in at least one allele.

When using the sgRNA3, bystander editing was also observed but was concentrated in the heart of the window activity. Preceding the nucleotide of interest, the C at position 3, which was the closest C of the On-Target position, underwent the most unwanted C>T editing (36.17%) (Figure 5B). Minor changes were also observed at the C in position 13, right outside the editing window, with 2.38% of C>T editing (Figure 5B). Once again, the on-target activity was greater, with more than 90% of the C>T ratio in at least one allele.

Overall, bystander editing was observed, but the percentage of on-target editing was much higher. Despite the number of chimeric clones, we were able to obtain multiple clones for each alteration with only the alterations of interest without bystander editing within the activity window of the cytidine deaminase.

### 3.5. Off-Target Mutagenesis

Apart from bystander editing, one of the major worries related to base editing for therapeutical applications or models’ creation concerns off-target editing. The risk of off-target alterations by CRISPR editing depends on the specificity of the target sequence. The more mismatches there are in the potential off-target sequence, the less possibility of off-target mutagenesis there will be. To assess the specificity of the CRISPR base editing, off-target cleavage activity was investigated at potential sites selected by the web-based prediction tool CRISPOR (http://crispor.tefor.net/, accessed on 17 September 2023). For each sgRNA, we selected the ten more likely off-target loci (Tables S3 and S4). We performed Sanger sequencing of these loci for two edited hiPSCs clones, one for each allelic state, together with the ancestor cell line for each *SH3TC2* variation. No off-target edits have been identified in any of the tested clones.

### 3.6. hiPSC Characterization

**Figure 6:**
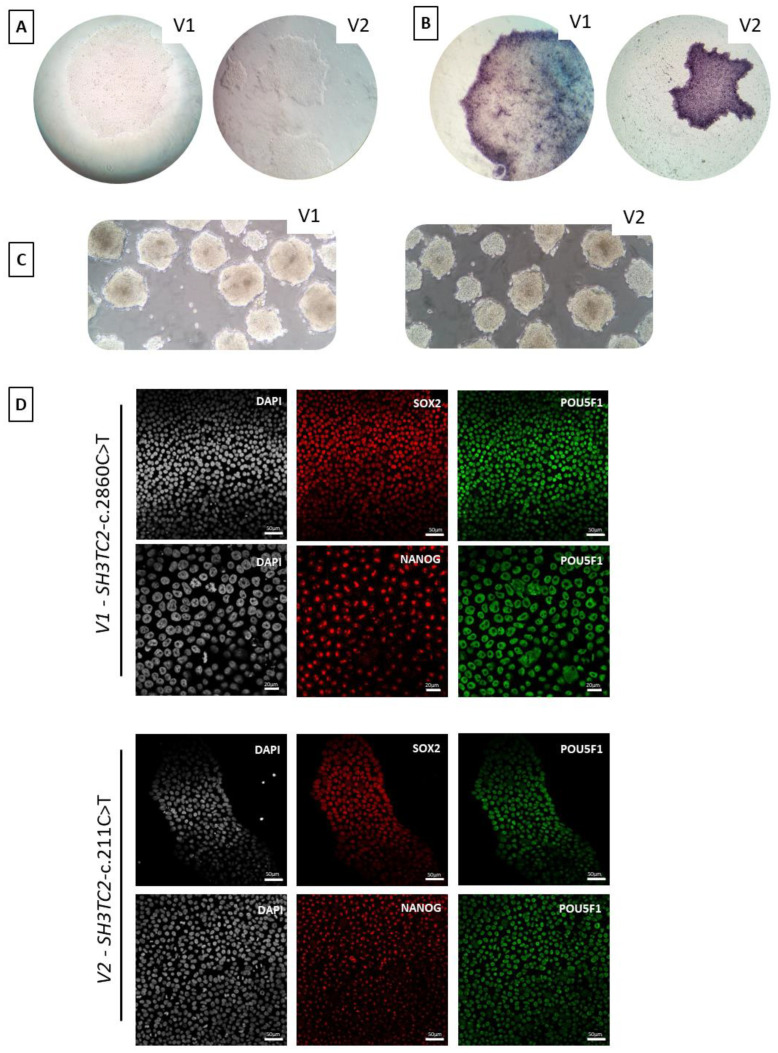
Morphology and characterization of both *SH3TC2*-c.2860C>T-p.Arg954* (V1) and *SH3TC2*-c.211C>G-p.Gln71* (V2) hiPSCs clones. (**A**) Morphology of hiPSCs clones displays a typical round-shaped colony morphology with small, tightly packed cells (objective ×100). (**B**) hiPSCs colonies expressing alkaline phosphatase (objective ×100). (**C**) Embryonic bodies formed spontaneously from hiPSCs clones (objective ×200). (**D**) Co-expression of pluripotency markers at protein level by immunofluorescence. Upper panel: SOX2 (red) and POU5F1 (green). Lower panel: NANOG (red) and POU5F1 (green). DAPI is represented in gray.

For each alteration, considering the recessive inheritance pattern of AR-CMTde-*SH3TC2*, we decided to characterize a homozygous hiPSCs clone. The selected clone for *SH3TC2*-c.2860C>T-p.Arg954* was named “V1” and the selected clone for *SH3TC2*-c.211C>T-p.Gln71* was named “V2”. Both selected clones presented morphological aspects of iPSCs (Figure 6A). Alkaline phosphatase expression of the clones (Figure 6B) and their ability to spontaneously form embryonic bodies were also assessed (Figure 6C). Additionally, immunocytochemistry experiments allowed us to check that the selected hiPSCs clone still expressed pluripotency markers, including NANOG, POU5F1, and SOX2 (Figure 6D). After these tests, we considered that the edited clones V1 and V2 were still pluripotent. The two selected clones also underwent array-CGH testing, which confirmed the absence of any genomic alterations caused by nucleofection (Figure S1).

## 4. Discussion

With the rise of disorders arising from genetic alteration, suitable models are heavily needed. In some cases, such as neuropathies, the limited access to affected cells in patients makes it arduous to study those disorders. The ability of hiPSCs to differentiate into the required cell type makes them an advantageous subject for generating appropriate cellular models, especially for neuroscience research. In addition, hiPSCs offer to work with cells sharing the same genetic and epigenetic background. Combined with hiPSCs, the CRISPR technologies eliminate the need for surgery encountered when generating hiPSCs from human fibroblasts, most often harvested by biopsies. Additionally, it allows for altering almost any part of the genome, generating clones faster than those starting from fibroblasts. In the last five years, differentiated CRISPR-engineered hiPS cellular models have greatly improved the study of human diseases [29,30,31].

One of the typical dilemmas encountered when performing classical CRISPR-Cas9 editing on hiPSCs using recombination is the limited efficiency of Homology Direct Repair (HDR), which typically yields editing efficiencies below 10%, often failing to reach even 1%. It should be noted that recent engineered versions of CRISPR, along with the consideration of alternative electroporation instruments and cell cycle phase, have improved HDR percentages to some extent, but editing efficiency remains relatively low. Besides efficiency, another major pitfall met when working with this type of CRISPR approach is its propensity to induce indels.

Recent advancements in base editing technology provide a valuable alternative for precise genomic base modifications in hiPSCs. Although, they do not entirely address the issue of non-target editing. Bystander editing is a common phenomenon observed in DNA when performing base editing [32]. Consequently, a wide range of improved base editors have been developed, offering various advantages, such as a narrowed editing window, high fidelity Cas9, and the ability to overcome the dependence on specific PAM sequences [11,12,33]. The choice of available BE has now greatly expanded, and therefore, it is difficult to establish the most suitable option and its compatibility with specific sgRNAs. Due to the time-consuming and expensive nature of growing and engineering hiPSCs, it becomes a considerable challenge to test multiple pairs of sgRNA-BE for each desired genetic alteration. By reviewing the literature, it is possible to choose the sgRNA-BE couple, which seems optimal. Unfortunately, given the recent progress regarding BE, there is currently limited data available to rely upon. Moreover, it is not always the appropriate combination that will prove to be the most effective in practice.

In this study, we have achieved a significant milestone by successfully generating the first two hiPSCs models for AR-CMTde-*SH3TC2* with remarkable efficiency. Utilizing the optimal strategy derived from our HEK-293T cell experiments, we aimed to develop robust AR-CMTde-*SH3TC2* models harboring either homozygous or heterozygous forms of the c.2860C>T or c.211C>T alterations in *SH3TC2*. The establishment of these novel hiPSCs models marks a significant breakthrough, holding tremendous potential for further advancing our understanding of the underlying pathogenesis of AR-CMTde-*SH3TC2* and towards the tests of targeted therapeutic approaches.

### 4.1. SpRY Base Editor Combined with sgRNA2 Allowed an Unexpected High Editing Efficiency

Recently, web tools created to compare various sgRNA-BE combinations have proved useful for their scope of prediction in silico [34,35]. However, such predictions can diverge significantly from observed editing at endogenous sites during in vitro experimentation [36].

For our experiments, from one combination to another, the results varied drastically. It appeared that combining either pCAG-CBE4max-SpRY-P2A-EGFP (SpRY), pCAG-CBE4max-SpG-P2A-EGFP (SpG) or YE1-BE4max-NG (YE1) BE with the sgRNA1 allowed observing a certain gradient of activity: SpRY>YE1>SpG, from highest to lowest efficiency. This result was quite unexpected. Indeed, with an “NGN” PAM such as the one comprised in the sgRNA1, it is recommended to use a SpG BE. This BE is known to have a similar to greater affinity with “NGN” PAM than SpRY [12,37]. Yet here, the other two tested BE allowed higher editing efficiency, up to 1.4 times higher using the SpRY rather than the SpG.

Several hypotheses can be issued to explain why the “predicted” pattern was not followed. For instance, the involvement in the editing efficiency of nucleotides located in the PAM site direct vicinity (PAM+1) has previously been demonstrated for the SpRY nuclease. As for SpG, it is known for promoting an even efficiency regardless of the surrounding nucleotides [12]. The PAM+1 motif present in the sgRNA1 is “NGNA”. It is the only combination that seems to promote similar or higher editing with SpRY. Also, note that different teams have faced “NGN” PAMs, wherein SpG was not able to edit when SpRY could [38,39].

Concerning the sgRNA2, SpRY was the only conceivable BE given the affiliated “AAG” PAM sequence. This combo allowed for better editing than the one observed with the sgRNA1. According to accepted rules used to identify optimal sgRNAs, the guide GC content was considered to be of primary importance. Higher GC content is associated with superior stability and should fall in the 40–80% range [40]. Here, sgRNA2 possessed a slightly higher GC content, with 40% (sgRNA1) and 45% (sgRNA2) GC ratio, respectively, which could possibly explain the divergence observed. Specific nucleotide positions are also known to hold a significant role in sgRNA binding efficiency. At the 17th position, A or T content was found in optimal sgRNA; this is the case in sgRNA1 but not in sgRNA2. In conclusion, none of the above previous data and guidelines could have helped to choose between sgRNA1 and sgRNA2, supporting the necessity of testing them in vitro in an appropriate model.

### 4.2. Editing Efficiency Divergence between HEK-293T and hiPSCs

Transfecting both the BE and the sgRNA in HEK-293T cells offered to edit approximately 30 to 60% of cells, an already considerable efficiency. However, almost 100% of the cells were found to be GFP+, and we could have expected an even higher editing ratio. Rather, on hiPSCs, a small portion of cells was correctly nucleofected. However, the GFP cell sorting allowed the selection of the nucleofected cells. Among the sorted hiPSCs, we obtained *SH3TC2*-c.211-p.Gln71* clones with an editing efficiency at the nucleotide of interest up to 93% and *SH3TC2*-c.2860-p.Arg954* clones have an editing efficiency at the nucleotide of interest of more than 90%, showing a very high efficiency of our strategy to obtain edited hiPSCs. One hypothesis that explains the higher editing efficiency in hiPSCs could be the DNA introduction method. Indeed, the nucleofection used on hiPSCs allows direct and quick DNA introduction into the nucleus, while with the CaCl_2_ transfection method, the plasmids enter the cytoplasm, and we rely on the NLS sequence comprised in the BE plasmid to address the base editing components from the cytoplasm to the nucleus. Other teams observed the same editing rate gap between HEK-293T cells and hiPSCs with higher editing in hiPSCs [41].

### 4.3. Base Editing outside the Activity Window and Consequences

Each base editor possesses a given activity window that can deviate from theory according to several criteria. The activity window for the SpRY base editor encompasses the nucleotide 4 to 8 bases 21 to 23 corresponding to the PAM sequence [12,42,43]. This window corresponds to the position of ssDNA uncovered by the protein-RNA-DNA ternary “R-loop” complex formed after the Cas9 binding to the DNA sequence. Within this DNA portion, while they are not the primary editing target, these nucleotides can be edited at lower efficiency by the cytidine deaminase enzyme. Such an event, named “bystander editing”, can affect the targeted sequence with varying risks depending, among others, on the favored motif of the deaminases. The deaminase that was comprised in all our BE was the APOBEC1. This enzyme preferentially targets cytidines preceded by thymine (T), which is the case for the cytosine at position 12th in the sgRNA2. Also, adenines (A) preceding the cytosine hindered the editing of such cytosine, which is also the case for the cytosine at position 9th in the sgRNA2 [44,45]. The sequence inclination of APOBEC1 could explain the editing preferences of the cytosine at the 12th position over the 9th one, even if this last one is closer to the activity window.

It is also important to oversee the possible fallout of such bystander editing. In this study, our major bystander editing resulted in a silent amino acid transition using the sgRNA2 for *SH3TC2*-c.2860C>T-p.Arg954*. Indeed, the editing of the cytosine at position 12 did not change the leucine amino acid (CTA > TTA). We have to be careful with such variation. Indeed, even if it is a silent amino acid, it could create or disrupt a splice site or other regulatory sequences. In addition, it was recently highlighted that synonymous alteration could still affect protein folding [46]. In our case, this variation has never been detected in the general population, meaning its consequences would be hard to assume. We therefore chose to eliminate those clones.

### 4.4. Towards Improved Neural Models to Study Neurogenerative Disorders

With the advancements in CRISPR-engineered hiPSC-based research, hiPSC neuronal differentiation emerges as a critical requirement to further understand neurodegenerative diseases. While traditional approaches relying on cell lines and animal models have provided crucial insights, the importance of more appropriate models cannot be overstated. By transitioning from generic cell lines and animal samples to hiPSC-derived neuronal cells, researchers gain a powerful tool that closely mimics the primary cell type affected, thereby enhancing the relevance and accuracy of their investigations. This approach not only enables a more comprehensive understanding of the disease-specific molecular and cellular mechanisms, bridging the gap between in vitro models and the complex pathophysiology observed in patients’ disease pathology but also facilitates the exploration of personalized medicine.

In the context of the Charcot–Marie–Tooth disease, various hiPSC models were generated in the past decade [47], but none for the most common recessive demyelinating form of the condition. Previous models were always reprogrammed from patients’ cells, and while few publications mentioned the use of the CRISPR technology, it was always employed to reverse a specific alteration in hiPSCs and obtain an isogenic clone [48,49,50], rather than to generate models. In contrast, our approach involved starting with a single healthy hiPSCs model, allowing us to create two different AR-CMTde-*SH3TC2* models that share the same genetic and epigenetic background as the control clone. A multitude of isogenic models could be generated with this technique, differing only by a single base, the origin of the disease.

## Data Availability

The raw data supporting the conclusions of this article will be made available by the authors on request.

## Supplementary Materials

The following supporting information can be downloaded at: https://www.mdpi.com/article/10.3390/biomedicines12071550/s1, Figure S1: Array Comparative Genomic Hybridization (aCGH) Analysis performed using G3 Human CGH microarrays 8 × 60 K (Agilent Technologies). Whole-genome idiograms of human induced pluripotent stem cell (hiPSC) clone (A) *SH3TC2*-c.2860C>T-pArg954* (V1) and (B) *SH3TC2*-c.211C>T-pGln71* (V2). These clones were hybridized and compared against the parental control clone (C) as the normal reference sample; Table S1: Base Editor and sgRNA plasmid combinations summary for the c.2860C>T; p.Arg954* editing of SH3TC2; Table S2: Base Editor and sgRNA plasmid combinations summary for the c.211C>T; p.Gln71* editing of SH3TC2; Table S3: Sequences of predicted genomic off-target loci containing two to four mismatches (MM), c.2860 C>T, p.Arg954*. The different off-target sites are ordered by number of mismatches and MIT score. The on-target sequence nucleotides are represented with a grey background. For all sequences, PAM sites nucleotides are colored in blue. For the on-target sequences those same nucleotides are underlined. For off-target sequences, nucleotides that vary from the on-target sequence are represented in bold with an orange background and in a blue background for the PAM site; Table S4: Sequences of predicted genomic off-target loci containing two to four mismatches (MM), c.211 C>T, p.Gln71*. The different off-target sites are ordered by number of mismatches and MIT score. The on-target sequence nucleotides are represented with a grey background. For all sequences, PAM sites nucleotides are colored in blue. For the on-target sequences those same nucleotides are underlined. For off target sequences, nucleotides that vary from the on-target sequence are represented in bold with an orange background and in a blue background for the PAM site; Table S5: Summary of the primers used for genotyping. A) Primers used for PCR and Sanger sequencing; B) Primers used for Allele-Specific PCR. The reference sequences for F primers are presented in order to better localize the variations present in the Forward WT primers and in the Forward Mutated primers, highlighted in red colour; Table S6: Summary of the primers used to sequence off-target c.2860C>T-p.Arg954*; Table S7: Summary of the primers used to sequence off-target c.211C>T-p.Gln71*; Table S8: Summary of the antibodies used for pluripotency characterization of hiPSCs.
