# Supplementary figures and images for: CRISPR Base Editing to Create Potential Charcot–Marie–Tooth Disease Models with High Editing Efficiency: Human Induced Pluripotent Stem Cell Harboring SH3TC2 Variants
Source: Biomedicines. 2024 Jul 12;12(7):1550. doi: 10.3390/biomedicines12071550 (PMC11274897; doi:10.3390/biomedicines12071550)

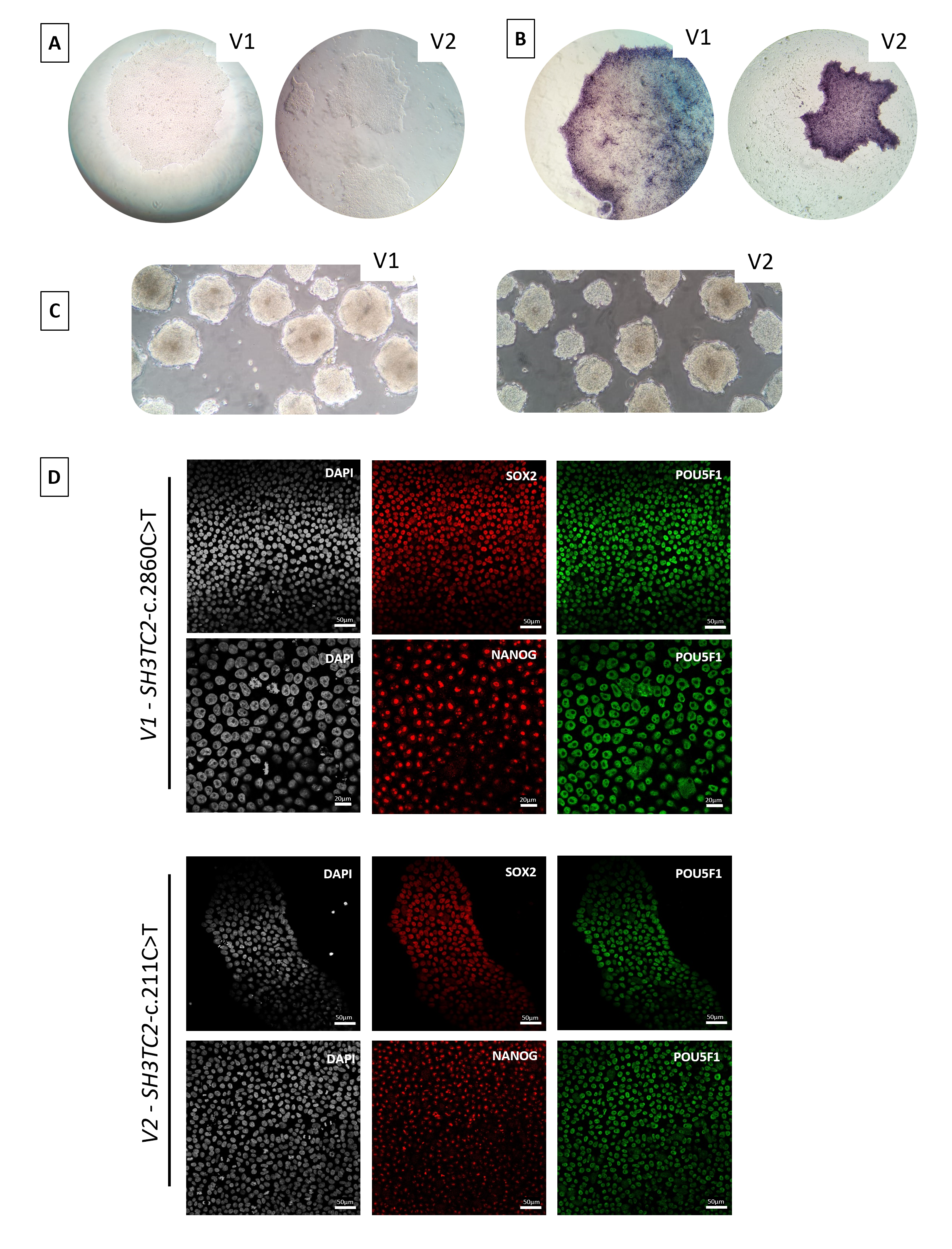

Supplement: Supplementary file 1 [file biomedicines-12-01550-s001.zip › Supplementary Figure 1.png]
