# Supplementary material for: CRISPR Base Editing to Create Potential Charcot–Marie–Tooth Disease Models with High Editing Efficiency: Human Induced Pluripotent Stem Cell Harboring SH3TC2 Variants
Source: Biomedicines. 2024 Jul 12;12(7):1550. doi: 10.3390/biomedicines12071550 (PMC11274897; doi:10.3390/biomedicines12071550)
Supplement: Supplementary file 3 [file biomedicines-12-01550-s001.zip › Supplementary Tables.odt]

Table S1.  Base Editor and sgRNA plasmid combinations summary for the c.2860C>T; p.Arg954* editing of SH3TC2  

| Guides | Guide sequence | PAM | Base editor | BE  accepted PAM | Window activity |
| --- | --- | --- | --- | --- | --- |
| sgRNA1 | GCATC5GACATCTAAAGAGTA | AGT | YE1   -  BE4max    -    NG | NGN | 1-2 |
| pCAG - CBE4max  -  SpG  -  P2A – EGFP | 4-8 |
| pCAG - CBE4max  -  SpRY -  P2A – EGFP | NNN |
| sgRNA2 | GGCATC6GACATCTAAAGAGT | AAG | pCAG - CBE4max  -  SpRY -  P2A – EGFP |

Table S2.  Base Editor and sgRNA plasmid combinations summary for the c.211C>T; p.Gln71* editing of SH3TC2  

| Guides | Guide sequence | PAM | Base editor | BE  accepted PAM | Window activity |
| --- | --- | --- | --- | --- | --- |
| sgRNA3 | CCCTAC6AGGAAGCTGCTCGG | AGG | YE1   -  BE4max    -    NG | NGN | 1-2 |
| pCAG - CBE4max  -  SpG  -  P2A – EGFP | 4-8 |
| pCAG - CBE4max  -  SpRY -  P2A – EGFP | NNN |

Table S3. Sequences of predicted genomic off-target loci containing two to four mismatches (MM), c.2860 C>T, p.Arg954*. The different off-target sites are ordered by number of mismatches and MIT score. The on-target sequence nucleotides are represented with a grey background. For all sequences, PAM sites nucleotides are colored in blue. For the on-target sequences those same nucleotides are underlined. For off-target sequences, nucleotides that vary from the on-target sequence are represented in bold with an orange background and in a blue background for the PAM site. 

| SH3TC2 | G | G | C | A | T | C | G | A | C | A | T | C | T | A | A | A | G | A | G | T | A | A | G |  |  |  |
| --- | --- | --- | --- | --- | --- | --- | --- | --- | --- | --- | --- | --- | --- | --- | --- | --- | --- | --- | --- | --- | --- | --- | --- | --- | --- | --- |
| Off-target | Sequence | MM | MIT Score | CFD Score |
| SLC7A3 | G | G | C | T | T | C | G | A | C | A | T | C | T | A | A | A | G | A | T | T | T | G | G | 2 | 4,27 | 0,42 |
| SLC39A9 | G | G | C | A | T | C | C | A | C | A | T | C | T | A | A | A | G | A | G | G | C | A | G | 2 | 3,15 | 0,03 |
| EBF1 | G | G | C | A | T | C | G | A | C | T | T | C | T | G | A | A | G | A | G | T | C | T | T | 2 | 0,83 | 0 |
| MYO9A | G | T | C | C | A | C | G | A | C | A | T | C | T | A | A | A | G | A | G | T | C | T | T | 3 | 2,32 | 0 |
| MYO18B | T | A | C | A | T | A | G | A | C | A | T | C | T | A | A | A | G | A | G | T | T | C | T | 3 | 1,47 | 0 |
| Y_RNA-CCNYL | A | G | C | T | T | C | A | A | G | A | T | C | T | A | A | A | G | A | G | T | T | G | G | 4 | 0,57 | 0,35 |
| PAX5 | A | G | C | A | T | A | G | A | A | A | G | C | T | A | A | A | G | A | G | T | T | G | G | 4 | 0,29 | 0,29 |
| MSI2 | A | G | C | A | T | C | A | A | C | A | C | G | T | A | A | A | G | A | G | T | A | G | G | 4 | 0,27 | 0,27 |
| NR1D2 | G | T | C | A | T | C | A | A | C | A | A | C | T | A | A | A | G | A | A | T | A | G | G | 4 | 0,19 | 0,34 |
| GRIA1 | G | G | C | A | A | C | A | A | C | A | T | C | C | A | A | A | A | A | G | T | G | G | G | 4 | 0,15 | 0,37 |

Table S4. Sequences of predicted genomic off-target loci containing two to four mismatches (MM), c.211 C>T, p.Gln71*. The different off-target sites are ordered by number of mismatches and MIT score. The on-target sequence nucleotides are represented with a grey background. For all sequences, PAM sites nucleotides are colored in blue. For the on-target sequences those same nucleotides are underlined. For off target sequences, nucleotides that vary from the on-target sequence are represented in bold with an orange background and in a blue background for the PAM site. 

| SH3TC2 | C | C | C | T | A | C | A | G | G | A | A | G | C | T | G | C | T | C | G | G | A | G | G |  |  |  |
| --- | --- | --- | --- | --- | --- | --- | --- | --- | --- | --- | --- | --- | --- | --- | --- | --- | --- | --- | --- | --- | --- | --- | --- | --- | --- | --- |
| Off-target | Sequence | MM | MIT Score | CFD Score |
| LGALS3BP | C | C | C | G | A | C | A | G | G | A | A | G | C | T | G | C | T | C | G | A | G | G | A | 2 | 1,28 | 0,04 |
| KRT8P47/RP5-1198O20.4 | A | C | C | T | A | C | A | G | G | A | A | G | C | T | G | C | T | A | G | A | G | G | G | 3 | 0,29 | 0,50 |
| GFAP | A | C | C | T | A | C | A | G | G | A | A | G | C | T | G | C | T | A | G | A | G | G | G | 3 | 0,29 | 0,50 |
| SMARCA4 | G | C | C | T | C | C | A | G | G | A | A | G | C | T | G | C | T | C | G | T | G | A | G | 3 | 0,30 | 0,08 |
| ARL3 | T | C | C | G | A | C | A | G | G | A | A | G | C | T | G | C | T | G | G | G | T | G | G | 3 | 0,66 | 0,08 |
| AC067959.1 | C | C | C | T | A | C | A | A | G | T | A | G | C | T | G | C | T | C | A | G | G | G | G | 3 | 0,82 | 0,63 |
| TRAPPC12 | C | C | A | G | C | C | T | G | G | A | A | G | C | T | G | C | T | C | G | G | T | G | G | 4 | 0,88 | 0,12 |
| PLEKHG4B | C | C | A | T | C | G | A | G | G | G | A | G | C | T | G | C | T | C | G | G | G | G | G | 4 | 0,75 | 0,12 |
| MTRNR2L13-TRMT112P1 | C | C | C | T | A | C | A | G | G | A | A | G | T | T | G | A | C | C | A | G | G | G | G | 4 | 0,011 | 0,352 |
| Y_RNA-MIR378C | A | C | A | G | A | C | A | T | G | A | A | G | C | T | G | C | T | C | G | G | T | G | G | 4 | 1,35 | 0,34 |

Table S5. Summary of the primers used for genotyping. A) Primers used for PCR and Sanger sequencing; B) Primers used for Allele-Specific PCR. The reference sequences for F primers are presented in order to better localize the variations present in the Forward WT primers and in the Forward Mutated primers, highlighted in red colour.

A)

| PCR and Sanger Sequencing |
| --- |
| Gene | Primer |
| SH3TC2-c.211C>T-p.Gln71* | Forward |  | CAGAGAAGCAGGGCATGAGC |  |
| Reverse |  | ACTGAGGACAGCTGATTGGC |  |
| SH3TC2-c.2860C>T-p.Arg954* | Forward |  | TAACCAGGCAGTGGCTATGG |  |
| Reverse |  | GGGCAGCTTTATCGGAACCT |  |

B)

Allele-Specific PCR

Variations

Sequences

Forward - Mut

Reverse

Forward - WT

Reference - F

SH3TC2

c.211C>T

p.Gln71* 

SH3TC2

c.2860C>T

p.Arg954* 

Forward - Mut

Reverse

Forward - WT

Reference - F

AGGGATCCTGTAGCAGACTGA

GGTGTGTAAATGGACCCTTAT

GGTGTGTAAATGGACCCTTAC

TGCTGTTTGGCTTAAGGGATT

CCTGAACACAAAGCAGGGAC

TGCTGTTTGGCTTAAGGGATC

TGCTGTTTGGCTTAAGGCATC

GGTGTGTAAATGGACCCCTAC

Table S6. Summary of the primers used to sequence off-target c.2860C>T-p.Arg954*  

| Off-target | Primer |
| --- | --- |
| SLC7A3 | Forward | CAGCTCTCTGGTAAGCGTTG |
| Reverse | CCTTGGCCTGTGGCTATTTG |
| SLC39A9 | Forward | CTTCTTGACTGGAGGAAGAG |
| Reverse | ATTGCGTCTCTTTGGTAGCC |
| PAX5 | Forward | ATGAGGGAAAGTTAGGAGAC |
| Reverse | TCCATGCCAACTATTTGGTG |
| MSI2 | Forward | TGACTGGCACAGTTTCGTGG |
| Reverse | TTCATTCCGATTCTGCCACC |
| NR1D2 | Forward | CCCTTTGGTCTCTGTATTGC |
| Reverse | CCTCCACTCCCTAATTAGAG |
| Y_RNA-CCNYL | Forward | GCAAGTTCCTAGTTTAATGGG |
| Reverse | AAAACTCTGAGCTCACAGGAG |
| EBF1 | Forward | GCAATTCGTGGGCATCTTTG |
| Reverse | CCAGTTGCTTGGAAAGACTC |
| MYO9A | Forward | GTGAGCTCTTACTGTGACTG |
| Reverse | CTCGGTAGAATGGAGATTTGG |
| MYO18B | Forward | GCAGGGAATATTCTCACCAC |
| Reverse | ACCTTGGAGGTAAAGTCTATG |
| GRIA1 | Forward | ACCCACCCAGAAACAGCAAC |
| Reverse | CAAGCCACCCTTCTATCTCC |

Table S7. Summary of the primers used to sequence off-target c.211C>T-p.Gln71*  

| Off-target | Primer |
| --- | --- |
| LGALS3BP | Forward | ACCTGGTTGAAAGGTCACAG |
| Reverse | GGTCACCAATCACCACTAAC |
| KRT8P47/RP5-1198O20.4 | Forward | AGACCTCCGAGATAAACTGG |
| Reverse | CAGGTCTGGATCTTCTTCAC |
| GFAP | Forward | ATCCCACAGAACGAGTCCC |
| Reverse | ATGTGTGAGGCAGGCACTG |
| SMARCA4 | Forward | TAACGTCTGCATCTCAGGAG |
| Reverse | TGCTTCTGTTTACCACGCTG |
| PLEKHG4B | Forward | ACCATCAGCTTGAGGAGTGG |
| Reverse | CAGAAGCTGGCTCCTTGATC |
| ARL3 | Forward | TTTAGACAATTCCAGTGGGC |
| Reverse | CTCCTTACATTCCAGATGTC |
| TRAPPC12 | Forward | AGGAACAGACCAAAGCCAGG |
| Reverse | GAAAGGGACACGACAGAATC |
| AC067959.1 | Forward | TGGGAAGAAAGGCACAGAGC |
| Reverse | GTGACCAAAAGTCCTTCTCC |
| MTRNR2L13-TRMT112P1 | Forward | AATAGTGGCCACATAGTGGC |
| Reverse | CTCTGTCAAGAAATACCGGAG |
| Y_RNA-MIR378C | Forward | CTAGAGCTCAGTCACATGAG |
| Reverse | CACATCCTGCCATCAAGCAA |

Table S8. Summary of the antibodies used for pluripotency characterization of hiPSCs

| Antibody | Supplier | Category | Dilution |
| --- | --- | --- | --- |
| NANOG | Abcam | Ab80892 | 1:100 |
| POU5F1 | Santa Cruz Biotech | Sc-5779 | 1:100 |
| SOX2 | Biolegend | 630802 | 1:200 |
